# Low ERCC1 expression is a good predictive marker in lung adenocarcinoma patients receiving chemotherapy based on platinum in all TNM stages - a single-center study

**DOI:** 10.1186/s13000-019-0885-2

**Published:** 2019-09-14

**Authors:** Marina Piljić Burazer, Suzana Mladinov, Antonela Matana, Sendi Kuret, Joško Bezić, Merica Glavina Durdov

**Affiliations:** 1 Institutet of Pathology, Forensic Medicine and Cytology, Clinical Hospital Center Split, Spinčićeva 1, 21000 Split, Croatia; 2 Department of Pulmonology, Clinical Hospital Center Split, Spinčićeva 1, 21000 Split, Croatia; 3 Department of Medical Biology, University of Split, School of Medicine, Šoltanska 2, 21000 Split, Croatia

**Keywords:** Lung adenocarcinoma, ERCC1, Platinum-based chemotherapy

## Abstract

**Background:**

High ERCC1 expression is thought to be related with resistance to chemotherapy based on platinum. The aim of this study was to present our institutional observations regarding to the association of ERCC1 and overall survival (OS) of the lung adenocarcinoma patients who received chemotherapy based on platinum.

**Material/methods:**

A total of 253 lung adenocarcinoma patients in all TNM stages were retrospectively investigated. The diagnosis was based on small biopsy samples obtained during bronchoscopy. Depending on the TNM stage of the disease and clinical condition, patients received only the chemotherapy based on platinum, or in combination with radiotherapy or surgery. Tissue sample for ERCC1 immunohistochemical analysis was sufficient in 129 patients. Low from high ERCC1 expression was separated by the semi-quantitative H-score median.

**Results:**

High ERCC1 expression was found in 47.3% patients, and was correlated with higher TNM (*p* = 0.021), tumor enlargement (*p* = 0.002), positive lymph nodes (*p* = 0.001), positive distant metastasis (*p* = 0.005), and higher relative risk of death (*p* < 0.001). Furthermore, significance association was observed for low ERCC1 expression and better performance status (ECOG) (*p* = 0.023). Longer OS was strongly associated with a low ERCC1 expression, not only in the group of patients in TNM stage I-III, who were treated with combination of chemotherapy with surgery or radiotherapy (*p* = 0.002), but also in the group of patients in TNM stage IV who received only chemotherapy based on platinum (*p* < 0.001), compared with the patients in the same TNM stage and high ERCC1 expression.

**Conclusions:**

ERCC1 expression in lung adenocarcinoma is a useful prognostic marker and moreover, a useful predictive marker in patients receiving chemotherapy based on platinum in all stages of the disease.

## Background

Adenocarcinoma is commonest non-small cell lung carcinoma (NSCLC), generally diagnosed in the advanced stage of the disease when it is not operable [1]. If we exclude adenocarcinomas with PD-L1 expression ≥50%, and those with *ALK, EGFR* and *ROS* mutations, the basic current care of advanced disease is chemotherapy based on platinum [2]. Drugs based on platinum form platinum-DNA adducts that obstruct cell replication and cause apoptosis. DNA damage could be repaired trough many biological processes and cause resistance to the platinum [3]. On the contrary to the immune and the molecular targeted therapies, no specific predictive marker is so fare available for platinum-based chemotherapy in lung adenocarcinoma. The best validated marker is the endonuclease excision repair cross-complementation group 1 (ERCC1), one of thirty enzymes of the nucleotide excision repair pathway. ERCC1 is the major enzyme that repairs DNA damage caused by the action of platinum, and is one of the possible factors that reduces the platinum effect [4]. The results of many studies on the significance of ERCC1 in lung adenocarcinoma are inconsistent. Previous retrospective studies and meta-analyses have correlated high ERCC1 expression with poor prognosis and platinum resistance [3, 5, 6], in a contrast to study of Booton et al. who did not find any correlation between the ERCC1 status and survival [7]. Sad et al. reported that longer survival was associated with low ERCC1 expression in lung adenocarcinoma patients who received chemotherapy based on platinum compared with those with high ERCC1 expression [8]. The same observation was presented in the study of Zhao et al. who found out that in advanced NSCLC, low ERCC1 expression indicates better prognosis and improved effectiveness for platinum-based chemotherapy [9]. In a contrary, Lee et al. concluded in a prospective ERCC1 trial (ET) that selecting chemotherapy by using a commercially available ERCC1 antibody did not prolong survival [10].

Therefore, the aim of the study was to present our single-institution experience on the importance of ERCC1 analysis in lung adenocarcinoma patients.

## Material and methods

### Patients characteristics

During three-year period (2013–2015), on small biopsies obtained during bronchoscopy, 253 new patients (161 males) with primary lung adenocarcinoma, median age 65 years (minimum-maximum: 44–91) were diagnosed at the Institute of Pathology, Forensic Medicine and Cytology, Clinical Hospital Center Split, Croatia. Clinical data of the patients were available at the Pulmonology Department. Clinical staging of the lung adenocarcinoma according to the 8th TNM staging system was determined by imaging radiologic techniques [11]. Patients received only chemotherapy based on platinum, or combined with surgery or radiotherapy depending on the TNM stage and clinical condition. Symptomatically treated patients and those treated only by surgery were not included in the study. All patients in our study had ALK and *EGFR* analysis of tumor tissue (ALK expression by IHC and *EGFR* mutation by PCR). These patients were also treated with platinum-based therapy, because in time when we conducted the study, tyrosine kinase inhibitor (TKI) therapy was not yet approved in Croatia. ROS analysis was not performed because it was introduced in routine practice in the March 2019. Survival was followed-up during period of 53 months, starting from the time of diagnosis till the time of death or May 2017. From the Registry of Mortality death data were obtained. This study was approved by Ethics Committee of the Hospital (500–03/15/01/42).

### Immunohistochemistry

From the archive of the Institute of Pathology, Forensic Medicine and Cytology the paraffin blocks of tumor tissue were obtained, and sectioned at 5 μm thickness. The expression of ERCC1 in the nucleus of tumor cells was performed using mouse monoclonal primary antibody, ready to use (4F9) (DACO, Glostrup, Denmark), stained on automatic stainer GX BenchMark (Ventana, Tuscon, Arizona) and visualised with Ventana DAB Detection kit iVieW (Ventana, Tuscon, Arizona). Tissue sample for ERCC1 immunohistochemical analysis was sufficient in 129 (50.7%) patients and interpretation of ERCC1 expression was performed according to Olaussen et al. [12]. Briefly, intensity of positive nuclear reaction (from 0 to 3) was multiplied by the percentage of positive cells (0 if =0%, 0.1 if 1–9%, 0.5 if 10–49%, and 1.0 if ≥50%) to obtain a semi-quantitative H score. The median of H scores was chosen for separating low from high levels of ERCC1 expression. Positive internal control was respiratory epithelium i.e. staining intensity 2. Olympus microscope BX 51 (Olympus, Tokyo, Japan) was used for analysis of ERCC1 expression at magnification of 400x by three independent pathologists who were blinded to all patients’ characteristics and survival status.

### Statistical analysis

The SPSS software 19 for Windows (Chicago, Illinois, USA) was used for statistical analysis and *p* < 0.05 was considered statistically significant for all tests. The differences between the categorical characteristics were calculated by the χ^2^ test. The numerical characteristics were analysed by the Kruskal-Wallis test and the Mann-Whitney test. Factors associated with overall survival were evaluated by logistic regression and Cox univariate and multivariate analyses. Kaplan-Meier method was used to calculate survival curves, and log-rank test to evaluate differences between them.

## Results

### Patients characteristics and ERCC1 status

Among 253 patients, 161 (63.6%) were males, 96 (86.5%) were smokers, 163 (69%) with TNM stage IV disease and a median age of 65 years (minimum-maximum: 44–91 years). A total of 6% patients had positive ALK expression and 10% had *EGFR* mutation. The significant correlation between ALK expression or *EGFR* mutations with observed variables were not found. The median value of all high scores for ERCC1 expression of 0.1 (0–3) was used to separate tumors with low ERCC1 expression (i.e. ≤ 0.1) from high ERCC1 expression (i.e. > 0.1). The majority of patients, 142 (77.2%) were treated with platinum-based chemotherapy alone, and in 26 (14.1%) patients, chemotherapy was combined with surgical resection and in 16 (8.7%) with radiotherapy (Table 1).

**Table 1:** Patients characteristics (*N* = 253)

Characteristics		N (%)
Age (years)	≤65	125 (49.4%)
Sex	Male	161 (63.6%)
Smoking status	Yes	96 (86.5%)
ECOG PS^a^	0	75 (52.9%)
	1	31 (21.8%)
	2	17 (12%)
	3	8 (5.6%)
	4	11 (7.7%)
TNM stage	I	9 (3.8%)
	II	28 (12%)
	III	34 (14.6%)
	IV	163 (69.6%)
Tumor size	T 1	22 (9.2%)
	T 2	71 (29.7%)
	T 3	69 (28.9%)
	T 4	77 (32.2%)
Lymph node	Positive	177 (75%)
Metastasis	Yes	163 (69.4%)
ERCC1	≤0.1	68 (52.7%)
	> 0.1	61 (47.3%)
Therapy^b^	C	142 (77.2%)
	C + S	26 (14.1%)
	C + R	16 (8.7%)

^a^*ECOG PS* Eastern Cooperative Oncology Group Performance Status

^b^*C* chemotherapy, *S* surgical resection, *R* radiotherapy

A total of 129 (50.7%) patients had a sufficient material in paraffin block for immunohistochemistry analysis for ERCC1. High ERCC1 expression was found in 61 (47.3%) patients and was strongly correlated with higher TMN (*p* = 0.021), tumor enlargement (*p* = 0.002), positive lymph nodes (*p* = 0.001), distant metastasis (*p* = 0.005). Furthermore, significant association was observed for low ERCC1 expression and better clinical condition (ECOG) for grouped ECOG stages 3 and 4 compared to others ECOG stages (*p* = 0.023) (Table 2).

**Table 2:** Patients characteristics according to the ERCC1 status (*N* = 129)

		ERCC1			
		≤0.1 (*N* = 68)	> 0.1 (*N* = 61)	χ^2^	p
Sex	Male	44 (65%)	42 (69%)	0.249	0.378
Smoking status	Yes	28 (82%)	31 (82%)	0.007	0.589
ECOG^a^	0	25 (61%)	16 (36%)	9.498	0.023
	1	10 (24%)	9 (21%)		
	2	4 (10%)	8 (18%)		
	3	2 (5%)	3 (7%)		
	4	0 (0%)	8 (18%)		
Tumor size	T 1	10 (15%)	2 (3%)	15.325	0.002
	T 2	25 (37%)	14 (23%)		
	T 3	18 (27%)	13 (22%)		
	T 4	14 (21%)	31 (52%)		
Lymph node	Positive	47 (69%)	54 (92%)	9.742	0.001
Metastasis	Yes	48 (71%)	55 (90%)	7.657	0.005
TNM stage^b^	I	2 (3%)	0 (0%)	7.690	0.021
	II	7 (10%)	3 (5%)		
	III	11 (16%)	3 (5%)		
	IV	48 (71%)	55 (90%)		

^a^ECOG was grouped as 0: 1: 2: (3 + 4)

^b^TNM stage was grouped as (I + II): III: IV

### Survival analysis

During follow-up period of 53 months, 186 (73.5%) patients died. The median overall survival was 10 months (SE:0.87; 95% CI:6–14). Among deceased patients, a higher rate of high ERCC1 expression was found, compared with patients who were alive (95% vs. 52%; *p* < 0.001).

**Fig. 1:**
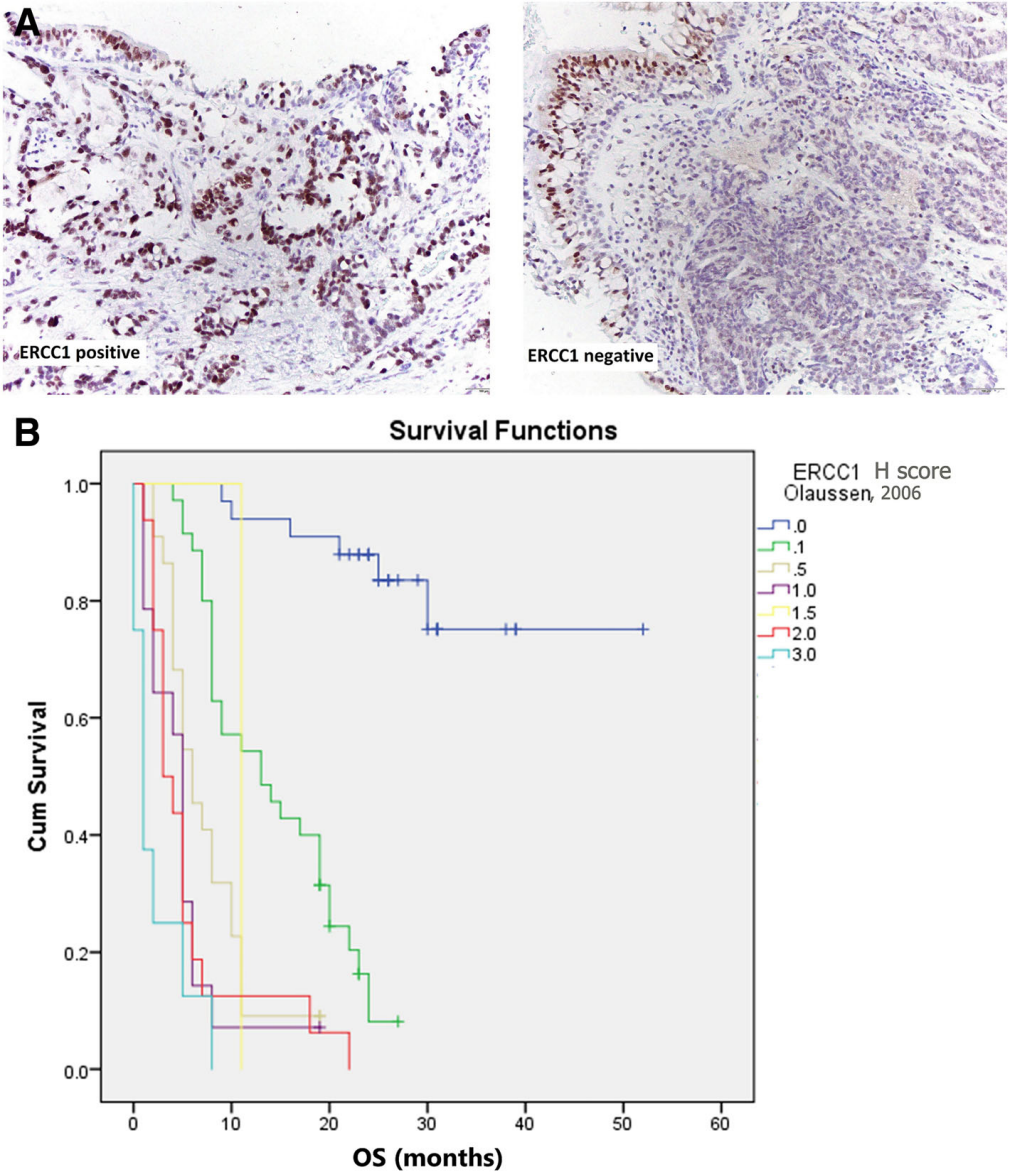
**a** ERCC1 immunohistochemistry in lung adenocarcinoma: positive and negative nuclear staining (HRP 200x). **b** Significant correlation was found between OS and level of ERCC1 expression (i.e. ERCC1 H scores, determined with multiplying the percentage of positive nuclear reaction by staining intensity). Longer OS was strongly associated with lower level of ERCC1 expression (lower H score) and a risk of death rose whenever ERCC1 expression rose (*p* < 0.001)

Univariate Cox’s regression analysis revealed factors that significantly influenced survival (Table 3). Higher relative risk for death was associated with high ERCC1 expression (*p* < 0.001), higher TNM and ECOG (*p* < 0.001; *p* < 0.001, respectively), tumor enlargement (*p* < 0.001), positive lymph nodes (*p* < 0.001) and distant metastasis (*p* < 0.001). Relative risk of death rose 2.491 times whenever ERCC1 expression rose (95%CI:2.019–3.073; *p* < 0.001) (Fig. 1a, b).

**Table 3:** Cox regression analysis for survival

		HR	95% CI	p
Sex	Male	0.803	0.594–1.086	0.154
Age (years)	≤65	1.063	0.797–1.417	0.678
Smoking status	Yes	1.110	0.573–2.153	0.757
ECOG	0–4	1.684	1.450–1.956	< 0.001
Tumor size	T1			< 0.001
	T2	1.336	0.649–2.750	
	T3	2.214	1.089–4.501	
	T4	4.445	2.212–8.935	
Lymph node	Positive	4.132	2.660–6.418	< 0.001
Metastasis	Yes	3.498	2.391–5.118	< 0.001
TNM stage^a^	I + II			< 0.001
	III	2.488	1.265–4.891	
	IV	5.474	3.107–9.645	
ERCC1	≤ 0.1	6.478	4.035–10.401	< 0.001
	> 0.1			

^a^TNM stage was grouped as (I + II): III: IV

Multivariate Cox’s regression analysis adjusted for significant prognostic factors for survival revealed high ERCC1 expression (HR:5.126; 95%CI:2.594–10.130; *p* < 0.001) and higher ECOG (HR:4.425; 95%CI:1.891–10.353; *p* = 0.008) for grouped ECOG stages 3 and 4 compared to others ECOG stages, as an independent factors of worse prognosis.

Statistically significant differences in overall survival were found according to ECOG (*p* < 0.001), tumor size (*p* < 0.001), lymph nodes (*p* < 0.001), distant metastasis (*p* < 0.001), TNM (*p* = 0.01), and ERCC1 expression (*p* < 0.001). OS was 5 times longer in patients with low ERCC1 expression compared with those with high ERCC1 expression (25 months vs. 5 months) (Table 4).

**Table 4:** Overall survival of lung adenocarcinoma patients according to the analysed variables

		Average survival (SE);95%CI	Median survival (SE);95%CI	LR	p
Age (years)	≤65	20.109 (1.762); 15.656–23.561	10.00 (0.972) 8.095–11.905	0.181	0.670
	> 65	19.331 (1.711); 15.977–22.684	10.00 (3.232) 3.664–16.336		
Sex	Male	18.443 (1529); 15.445–21.440	9 (1.002); 7.037–10.963	2.150	0.143
	Female	22.049 (2.049); 18.044–26.055	17 (3972); 9.215–24.785		
Smoking status	Yes	17.4 (1.935) 13.607–21.193	8 (1224) 5.600–10.400	0.102	0.750
	No	15.116 8.760–21.471	16 (10.797) 0–37.163		
ECOG	0	25.916 (2.468) 21.079–30.754	23 (3.910) 15.336–30.664	62.106	< 0.001
	1	12.172 (2.073) 8.109–16.235	7 (1.113) 4.819–9.181		
	2	10.882 (3.096) 4.815–16.950	7 (1.543) 3.975–10.025		
	3	4.750 (2.169) 0.498–9.002	2 (0.685) 0.658–3.342		
	4	2.36 (1.073) 0.534–4.739	2 (0.591) 0.842–3.158		
TNM stage*	I + II	39.355 (2.654) 34.154–44.556	39 (7.649) 24.009–53.991	50.830	< 0.001
	III	25.660 (3.171) 19.444–31.876	23 (3.312) 16.08–29.492		
	IV	13.861 (1.355)	7 (0.762)		
Tumor size	T1	32.709 (4702) 23.494–41.924		52.961	< 0.001
	T2	28.521 (2.419) 23.779–33.262	23 (3.512) 16.116–29.884		
	T3	19.520 (2.272) 15.068–23.973	10 (2.371) 2.371–5.353		
	T4	9.240 (1.201) 6.886–11.595	5 (0,625) 3.774–6.226		
Lymph node	Negative	36.468 (2.402) 31.761–41.175	45 (9468) 26.443–63.557	48.562	< 0.001
	Positive	12.137–17.275	7 (0,739) 5.552–8.448		
Metastasis	No	33.370 (2.201) 29.057–37.683	32 (5,4) 21.416–42.84	48.784	< 0.001
	Yes	13.819 (1.348) 11.178–16.461	7 (0,734) 5.561–8.439		
ERCC1	≤ 0.1	30.793 (2.543) 25.810–35.777	25 (4.594) 15.996–34.004	78.710	< 0.001
	> 0.1	5.951 (0.696) 4.586–7.316	5 (0.441) 4.135–5.865		
TNM IV/C**	≤ 0.1	24.719 (2.034) 20.732–28.705	24 (3.607) 16.931–31.069	65.412	< 0.001
	> 0.1	5.511 (0.564) 4.404–6.617	5 (0.295) 4.421–5.572		
TNM I-III/ C + S or R**	≤ 0.1	45.727 (4.025) 37.838–53.616		9.290	0.002
	> 0.1	11.400 (4.614) 2.356–20.444	8 (4.382) 0–18.588		

*TNM stage was grouped as (I + II): III: IV

**TNM IV/ C - TNM stage IV patients, receiving only chemotherapy based on platinum (C)

***TNM I-III/C + S or R - TNM stage I-III patients receiving combination of radiotherapy (R) or surgery (S) with chemotherapy (C)

**Fig. 2:**
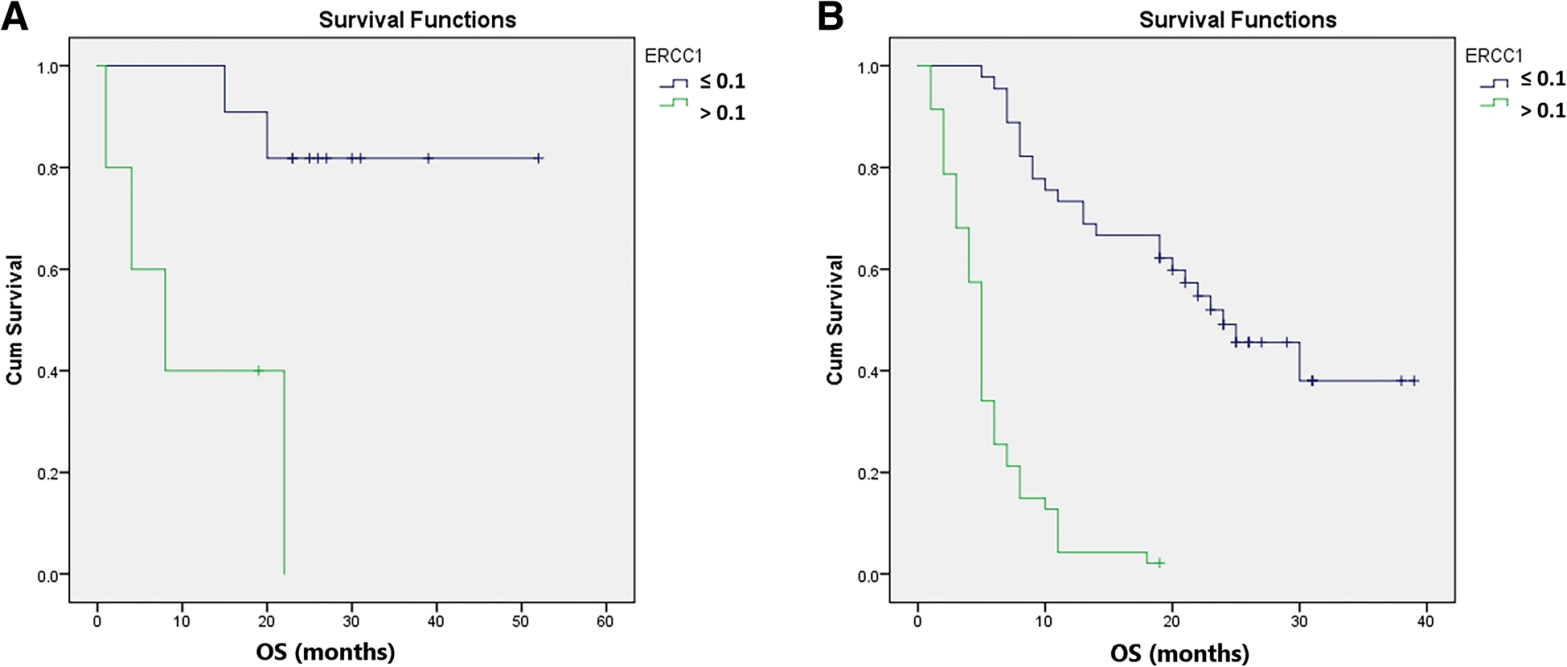
Association between the OS of the lung adenocarcinoma patients in different TNM stages/ treated with different therapeutic modalities, with ERCC1 expression. **a** Longer OS of the patients with TNM stage I – III/ treated with chemotherapy combined with radiotherapy or surgery, was associated with the low ERCC1 expression compared to the same TNM staged patients and high ERCC1 expression (*p* = 0.002). **b** Among the patients in TNM stage IV/treated only with chemotherapy based on platinum, those who had low ERCC1 expression in tumor, had longer OS compared with the same TNM staged patients and high ERCC1 expression (*p* < 0,001)

The predictive value of ERCC1 was analysed by observing the correlation between OS of the lung adenocarcinoma patients in different TNM stages treated with different therapeutic modalities, and ERCC1 status (Fig. 2a, b). Among 102 patients in TNM stage IV/ treated only with chemotherapy, low ERCC1 expression was found in 45 (44.12%) patients and was correlated with longer OS compared with those who had high ERCC1 expression (24 months vs. 5 months; *p* < 0.001). Furthermore, 27 patients were in TNM stages I-III and received chemotherapy based on platinum combined with surgery or radiotherapy. Low ERCC1 expression was found in 8 (29.6%) of these patients and was correlated with longer OS compared to those with high ERCC1 expression (*p* = 0.002).

## Discussion

Previous retrospective studies and meta-analyses revealed that high ERCC1 expression is a negative predictor in patients who received chemotherapy based on platinum and good prognostic marker in patients who did not [2, 3, 6, 13–15]. The same observations were found in ovarian epithelial carcinoma, squamous carcinoma of the head and neck, and gastroenteric carcinomas [16, 17]. So far, conflicting results were found in few studies according to correlation of ERCC1with prediction or prognosis in NSCLC. Findings in the meta-analysis of Roth et al. supported the hypothesis that ERCC1 is associated with response to chemotherapy based on platinum and OS in advanced NSCLC, but the study of Booton et al. did not confirmed that hypothesis [7, 14]. Several prospective studies have suggested that ERCC1 is predictive in NSCLC. The study by Cobo et al. prospectively evaluated expression of ERCC1 mRNA in advanced stage of NSCLC in order to predict response to cisplatin-free or cisplatin-based therapy. Patients in control group received docetaxel/cisplatin combination. The other group of patients received docetaxel/cisplatin or docetaxel/gemcitabine regimens respectively, according to low or high ERCC1 mRNA levels and their response rates were significantly higher (50.3%) compared with the control group (39.3%) [18]. Takemoto et al. prospectively evaluated the benefit of therapy without platinum for patients in TNM stages IIIB/IV who expressed high level of ERCC1 mRNA, and concluded that therapy without platinum might be effective for those patients [19]. In a contrast to these findings, Lee et al. concluded in a prospective ERCC1 trial (ET) that in patients with advanced disease, choosing the best suitable chemotherapy by using a commercially available ERCC1 antibody, did not prolong survival [10]. Our study was retrospective, and chemotherapy based on platinum was applied to all of our patients. In a few patients, chemotherapy was combined with surgery or radiotherapy depended on the TNM stage of the disease and the clinical condition of the patient. A correlation between low ERCC1 expression and longer OS was found in patients in all TMN stages. Knowing that ERCC1 is the major enzyme involved in platinum damage repair, this result was hypothetically expected, as a consequence of the ineffectiveness of the ERCC1 enzyme to repair the damage caused by platinum [20]. Awareness of the fact that a lot of other factors, in addition to ERCC1 contribute to chemotherapy response, more analysis are required for verification of this hypothesis.

ERCC1 status could be evaluated trough the immunohistochemical (IHC) staining or by real-time polymerase chain reaction (RT-PCR) method. Carter et al. emphasized the importance of selected therapy trough molecular analysis of cancer cells using IHC, with the intention to increase patients’ benefits, and ERCC1 was evaluated as a predictor of better outcome in lung adenocarcinoma patients [21]. In our study, the expression of ERCC1 was assessed trough IHC staining and graded using the same scoring system as Olaussen. The median H score of 0.1 (0–3) was used to define low versus high ERCC1 expression [5], and it was lower than in Olaussens study (0.1 vs 1), but when we correlated our results to patients’ outcome, the results between these two studies were in agreement. Although, IHC is useful method for evaluation the protein expression in tumor tissue, we have to be careful with the interpretation of IHC staining. There are several limitations such as different antibodies, variations among observers and the different values for separating low from high ERCC1 expression. This suggests that inconsistent prognostic interpretations of ERCC1 might have been influenced by the scoring system which emphasis the need for consensus about methodology [22]. Furthermore, the ERCC1 evaluation by IHC is questionable, because ERCC1 is present as four isoforms in the nucleus, but only one of them (isoform 202) was involved in the repair of platinum adducts. [23]. These observations could have been one of the possible explanation for discrepancy among the studies. Currently, none of the available antibody, allows for differentiating between four ERCC1 isoforms, leading to possibility of wrong evaluation and classification of ERCC1 expression. More valid method for ERCC1 analysis is RT-PCR for detection of the mRNA from tumor tissue. Prospective studies, mentioned above were conducted using paraffin blocks of tumor biopsies, previously fixed in formalin for mRNA extraction. Formalin destroys the mRNA, so tumor samples should be taken for ERCC1 analysis before fixation to avoid wrong evaluation of ERCC1 expression [19].

We assumed that the small sample tissue, as a commonest, and derived from bronchial biopsies, would be sufficient for IHC analysis of ERCC1 expression and representative for the total tumor despite of tumor heterogeneity, according to Taillade et al. who reported a high correlation for ERCC1 expression comparing bronchial biopsies and resected surgical specimens in the same patient [24].

In our research, beside ERCC1, a smaller tumor size was found out to be a factor of better prognosis which correlates with the findings of Rami-Porta et al. [25]. They emphasized the importance of the new 8thTNM staging system where T1 lesions were subdivided into T1a, T1b, and T1c lesions corresponding to lung cancers up to 10 mm, between 11 and 20 mm, and between 21 and 30 mm, respectively [10]. Prognosis was significantly better for the smallest lesions, as 5-year survival rates for clinical staging were 92, 83, and 76% for T1a, T1b, and T1c cancers, respectively [25]. It is obvious that tumour size is an important prognostic factor.

The prognosis of NSCLC has been changed with immunotherapy but without the long-term benefit for many patients. The association of ERCC1 with immunotherapy response was analysed, assuming that ineffective ERCC1 increases defects in the DNA of tumor cells and causes stronger immune response to the tumor. Chabanon et al. revealed correlation between low expression of ERCC1 and better response to immunotherapy due to increased neo-antigens in tumor cells [26]. It seems that ERCC1 has remained not only an important predictive marker of chemotherapy based on platinum, but it could also be a predictor of immunotherapy response.

## Conclusion

Our data indicated that high ERCC1 expression is a valuable negative prognostic marker in patients with lung adenocarcinoma. Novelty of the present study is that low ERCC1 expression is confirmed as a good predictive marker in all stages of lung adenocarcinoma in patients treated with platinum-based chemotherapy alone, or in combination with surgery or radiotherapy. IHC is a valuable method to evaluate the ERCC1 status, but consensus on the selection of most reliable antibody has to be achieved and values for separating low from high ERCC1 expression have to be established to avoid wrong classification of ERCC1 status. In the century of immunotherapy, ERCC1 is recognised as predictor of better response to check point inhibitors. Additional efforts have to be made to determine predictive markers to any therapeutic modality which could result in patients benefit.

## Data Availability

All dana generated or analysed during this study are included in this published article.

## Abbreviations

ALK: Anaplastic lymphoma kinase
C: Chemotherapy
ECOG PS: Eastern Cooperative; Oncology Group Performance Status
EGFR: Epidermal growth factor receptor
ERCC1: Excision repair cross-complementing group 1
HR: Hazard ratio
HRP: High resolving power
IHC: Immunohistochemistry
mRNA: Messenger ribonucleic acid
NSCLC: Non-small cell lung carcinoma
OS: Overal survivall
R: Radiotherapy
ROS: C-ros oncogene 1
S: Surgery
SPSS: Statistical Package for the Social Sciences
TNM: Tumor Nodes Methastasis

## References

[1] König K, Peifer M, Fassunke J (2015). Implementation of Amplicon Parallel Sequencing Leads to Improvement of Diagnosis and Therapy of Lung Cancer Patients. J Thorac Oncol.

[2] Olaussen KA, Postel VS (2016). Predictors of chemotherapy efficacy in non-small-cell lung cancer: a challenging landscape. Ann Oncol.

[3] Hubner RA, Riley RD, Billingham LJ (2011). Excision repair cross-complementation group 1 (ERCC1) status and lung cancer outcomes: a meta-analysis of published studies and recommendations. PLoS One.

[4] Martin LP, Hamilton TC, Schilder RJ (2008). Platinum resistance: the role of DNA repair pathways. Clin Cancer Res.

[5] Olaussen KA, Dunat A, Fouret P (2016). DNA repair by ERCC1 in non-small cell lung cancer and cisplatin-based adjuvant chemotherapy. N Engl J Med.

[6] Han Y, Lui J, Sun M (2016). A significant statistical advancement on the predictive values of ERCC1 polymorphisms for clinical outcomes of platinum-based chemotherapy in non-small cell lung carcinoma: an update meta-analysis. Dis Markers.

[7] Booton R, Ward T, Ashcroft L (2007). ERCC1 mRNA expression is not associated with response and survival after platinum-based chemotherapy regimens in advanced non-small cell lung cancer. J Thorc Oncol.

[8] Sad LM, Younis SG, Elity MM (2014). Prognostic and predictive role of ERCC1 protein expression in locally advanced stage III non-small cell lung cancer. Med Oncol.

[9] Zhao H, Zhabg H, Du Y (2014). Prognostic significance of BRCA 1, ERCC1, RRM1 and RRM1 in patients with advanced non-small cell lung carcinoma receiving chemotherapy. Tumour Biol.

[10] Lee SM, Falzon M, Blackhall F (2017). Randomized prospective biomarker trial of ERCC1 for comparing platinum and non-platinum therapy in advanced non-small-cell lung cancer: ERCC1 trial (ET). J Clin Oncol.

[11] Amin BM, editor. AJCC Cancer Staging Manual 8th edition. Springer, 2017;447–449.

[12] Olaussen KA, Dunant A, Fouret P (2006). DNA repair by ERCC1 in non-small-cell lung cancer and cisplatin-based adjuvant chemotherapy. N Engl J Med.

[13] Besse B, Olaussen KA, Soria JC (2013). ERCC1 and RRM1: ready for prime time?. J Clin Oncol.

[14] Roth JA, Carlson JJ (2011). Prognostic role of ERCC1 in advanced non-small-cell lung cancer: a systematic review and meta-analysis. Clin Lung Cancer.

[15] Ryu JS, Memon A, Lee SK (2014). ERCC1 and personalized medicine in lung cancer. Ann Transl Med.

[16] Kuhlmann JD, Wimberger P, Bankfalvi A (2014). ERCC1-positive circulating tumor cells in the blood of ovarian cancer patients as a predictive biomarker for platinum resistance. Clin Chem.

[17] Prochnow Sebastian, Wilczak W., Bosch V., Clauditz T. S., Muenscher A. (2018). ERCC1, XPF and XPA—locoregional differences and prognostic value of DNA repair protein expression in patients with head and neck squamous cell carcinoma. Clinical Oral Investigations.

[18] Cobo M, Isla D, Massuti B (2007). Customizing cisplatin based on quantitative excision repair cross-complementing 1 mRNA expression: a phase III trial in non-small-cell lung cancer. J Clin Oncol.

[19] Takemoto S, Nakamura Y, Gyoutoku H (2019). Phase II trial of a non-platinum triplet for patients with advanced non-small cell lung carcinoma (NSCLC) overexpressing ERCC1 messenger RNA. Thorac Cancer..

[20] Piljic Burazer M, Mladinov S, Ćapkun V (2017). The utility of thytoid transcription factor 1 (TTF-1), Napsin a, excision repair cross-complementing 1 (ERCC1), anaplastic lymphoma kinase (ALK) and the epidermal growth factor receptor (EGFR) expression in small biopsy in prognosis of patients with lung adenocarcinoma – a retrograde single-center study from Croatia. Med Sci Mont.

[21] Carter P, Alifrangis C, Cereser B (2018). Does molecular profiling of tumors using the Caris molecular intelligence platform improve outcomes for cancer patients?. Oncotarget.

[22] Ozdemir O, Ozdemir P, Veral A (2013). ERCC1 expression does not predict survival and treatment response in advanced stage non-small cell lung cancer cases treated with platinum based chemotherapy. Asian Pac J Cancer Prev.

[23] Postel-Vinay S, Soria JC (2017). ERCC1 as Predictor of Platinum Benefit in Non-Small-Cell Lung Cancer. J Clin Oncol.

[24] Taillade L, Penault-Llorca F, Boulet T (2007). Immunohistochemical expression of biomarkers: a comparative study between diagnostic bronchial biopsies and surgical specimens of non-small-cell lung cancer. Ann Oncol.

[25] Rami-Porta R, Bolejack V, Crowley J (2015). The IASLC lung Cancer staging project: proposals for the revisions of the T descriptors in the forthcoming eighth edition of the TNM classification for lung Cancer. J Thorac Oncol.

[26] Chabanon RM, Lord JC, Postrl-Vinay S (2019). PARP inhibition enhances tumor cell-intrinsic immunity in ERCC1 –deficient non-small cell lung cancer. J Clin Invest.

